# Sliding‐mode‐based controllers for automation of blood glucose concentration for type 1 diabetes

**DOI:** 10.1049/syb2.12015

**Published:** 2021-03-29

**Authors:** Sheraz Ahmad Babar, Iftikhar Ahmad, Iqra Shafeeq Mughal

**Affiliations:** ^1^ School of Electrical Engineering and Computer Science (SEECS) National University of Sciences and Technology (NUST) Islamabad Pakistan

## Abstract

Destruction of *β*‐cells in pancreas causes deficiency in insulin production that leads to diabetes in the human body. To cope with this problem, insulin is either taken orally during the day or injected into the patient's body using artificial pancreas (AP) during sleeping hours. Some mathematical models indicate that AP uses control algorithms to regulate blood glucose concentration (BGC). The extended Bergman minimal model (EBMM) incorporates, as a state variable, the disturbance in insulin level during medication due to either meal intake or burning sugar by engaging in physical exercise. In this research work, EBMM and proposed finite time robust controllers are used, including the sliding mode controller (SMC), backstepping SMC (BSMC) and supertwisting SMC (second‐order SMC or SOSMC) for automatic stabilisation of BGC in type 1 diabetic patients. The proposed SOSMC diminishes the chattering phenomenon which appears in the conventional SMC. The proposed BSMC is a recursive technique which becomes robust by the addition of the SMC. Lyapunov theory has been used to prove the asymptotic stability of the proposed controllers. Simulations have been carried out in MATLAB/Simulink for the comparative study of the proposed controllers under varying data of six different type 1 diabetic patients available in the literature.

## 1 INTRODUCTION

Diabetes is one of the most persistent diseases to evolve from numerous underlying processes in the human body. Diabetes mellitus belongs to the group of metabolic diseases that occur because of inadequate amounts of insulin to burn sugar, impaired insulin functioning or both. There are two categories of diabetes mellitus; type 1 diabetes, also known as insulin‐dependent diabetes, which accounts for ≃5–10 per cent of the incidence of diabetes within the human population, is caused by the destruction of beta cells in pancreas or the failure of insulin excretion, resulting in hyperglycaemia. On the other hand, when the blood glucose concentration (BGC) falls below the normal range, it is known as hypoglycaemia. Type 2 diabetes, which is non‐insulin dependent, accounts for ≃90–95 per cent of the incidence of diabetes within the human population. It is caused by a chronic condition in which the glucose level builds up within the bloodstream due to abnormalities in insulin function [1].

Diabetes with hyperglycaemia leads to deep‐rooted damage to nerves, kidneys, blood vessels and heart and may lead to the failure of some other organs, while hypoglycaemia diabetes can cause confusion, shakiness or drowsiness [2]. Diabetes is not only a life‐threatening disease but also an exponentially increasing burden on the economy. Every year, billions of dollars (US) are spent towards its cure. According to an economic survey, in 2002 about 132 billion US dollars, and in 2012 around 245 billion US dollars, were spent towards the cure for diabetes [3, 4]. In Hungary, the predominance of diabetes mellitus has increased over the years domestically and has hurt the economy badly. According to the World Health Organization (WHO), such an economic burden can be reduced by taking necessary action towards its cure [5].

The normal range of BGC for a healthy person is 70–130 mg/dl. The BGC of a type 1 diabetic patient should be monitored continuously and be brought down to the safe range. In 2012, the WHO reported that around 1.5 million deaths were caused by diabetes mellitus [6]. According to a predictive study, the current number of diabetic patients may cross 300 million by 2025 [7]. Diabetes mellitus threatens a life every 8 s and the loss of a limb every 30 s. Monitoring BGC during daytime is easy but is not possible during night‐time. To overcome this problem, artificial pancreas (AP) is the solution [8, 9].

Designing a controller for AP has always been a challenging task because of variable meal disturbances during medication. These disturbances can be caused by meal intake or by burning sugar during and after physical exercise. AP is a closed‐loop feedback system comprising three parts: sensor, controller and insulin pump. BGC is measured with the help of sensors, and the controller calculates the required amount of insulin to feed into the patient's body, and then the insulin pump injects the required amount of insulin into the body [10, 11]. The controller maintains the sugar‐insulin level at a stable reference level within the safe range.

For automatic regulation of BGC in type 1 diabetes, different algorithms, both linear and non‐linear, have been proposed in the literature. A linear quadratic Gaussian controller combined with insulin on board as a constraint, called the automatic regulation of glucose algorithm, has been proposed in [12]. Linear parameter‐varying is a model to design control inputs for AP [13]. The linear quadratic regulator algorithm has also been proposed to design a controller for type 1 diabetic patients in [14]. The conventional proportional–integral–derivative (PID) controller has been proposed to control the blood glucose level to achieve reduced steady‐state error [15]. The oscillations that appear in the response of the PID controller have been eliminated by the proportional derivative controller [16]. The fuzzy controller has been implemented in the literature with better results but is computationally very costly [17, 18]. To design linear controllers, linearised models must ensure local stability, as non‐linear terms may be neglected only in a certain region very close to the point of operation, whereas non‐linear controllers do not need linearisation for their design and therefore can talk globally. They perform quite a bit better even in the presence of model variations, uncertainties, external disturbances and non‐linearities. Among those using the extended Bergman minimal model (EBMM), SMC has been proposed in [19] to achieve robustness and the required design specification of BGC but has an inherent chattering phenomenon in its response. In the second‐order sliding mode controller (SOSMC), real and supertwisting algorithms have been introduced that steer system trajectories in the vicinity of the sliding surface to obtain finite time convergence. SOSMC algorithms have the advantages of insensibility to perturbations and reduced chattering, which reflect their high convergence accuracy and robustness [20, 21]. The backstepping (BS) controller has also been proposed to stabilise systems that have a strict feedback form [22]. To improve the results for BGC given by BS, the addition of an adaptive parameter has been proposed that has better convergence time but overshoots/undershoots [23, 24].

The dynamics of diabetes mellitus type 1 are non‐linear. In this paper, we have proposed three non‐linear‐based controllers including the SMC, supertwisting SOSMC and backstepping sliding mode controller (BSMC) for regulation of BGC in type 1 diabetes through AP with the complete mathematical derivation of each proposed controller. Simulation results have been presented using MATLAB/Simulink to check the comparative performance of the proposed controllers. From information available in the literature, we have simulated data for six different type 1 diabetic patients using supertwisting SOSMC.

Salient features of this research paper are listed below:

- The key challenges are to develop robust controllers to achieve better settling and convergence time with reduced steady‐state error.
- Three robust non‐linear controllers have been proposed to accommodate the effects of non‐linearities and variable meal disturbance present in the system.
- The conventional SMC has been proposed for robustness, but it exhibits an inherent chattering phenomenon.
- The supertwisting SOSMC has been proposed to achieve an even better tracking response that reduces the chattering effect.
- SMC has been merged with the BS algorithm, which makes the controller robust to achieve the desired reference level quite nicely.
- Stability analysis for each proposed controller has been proved with the help of Lyapunov stability theory.
- Performance comparisons of the proposed controllers has been made to deduce the outperforming controller among those that have been proposed.
- Perturbation as Gaussian noise *d*(*t*) has been added in the system, and the output performance of each has been analysed to check their robustness.
- The proposed supertwisting SOSMC has also been analysed using the varied data available in the literature of six type 1 diabetic patients.

The rest of the paper is organised as follows. The blood sugar regulation system, non‐linear mathematical model and problem statement are explained in Section 2. Section 3 describes the analysis and design of the proposed non‐linear controllers for AP using the EBMM. Section 4 details all simulation results, and finally, Section 5 concludes.

## 2 NON‐LINEAR EXTENDED BERGMAN MINIMAL MODEL FOR TYPE 1 DIABETIC PATIENTS

### 2.1 Blood sugar regulation system

**FIGURE 1:**
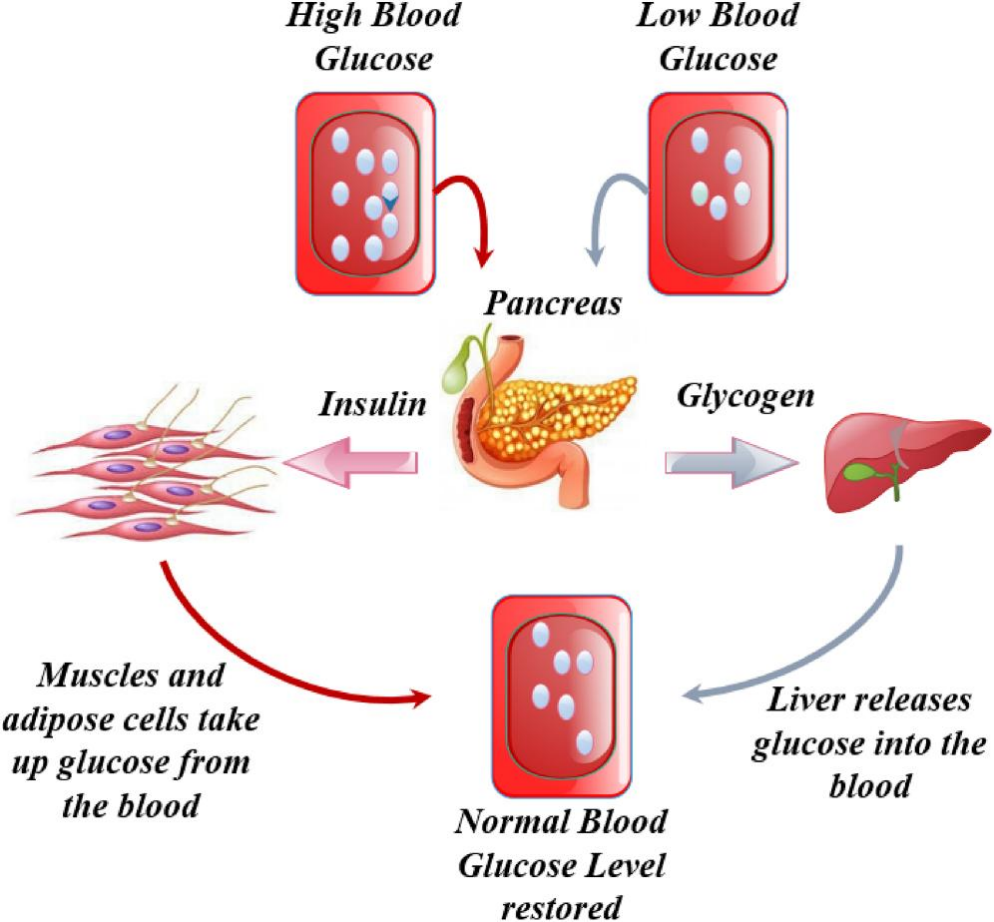
Pancreas controlling blood glucose level in the human body

Secretion of insulin and glycogen plays an important role in the regulation of blood sugar (glucose) within the human body. BGC is considered normal when insulin and glycogen sustain a state called ‘homeostasis’. When the BGC rises above the normal range of 70–120 mg/dl, the pancreas secretes insulin to burn excessive sugar. On the other hand, when the BGC falls below the normal range, glycogen is released by the pancreas to increase the glucose level in the blood as shown by Figure 1. This balanced functioning prevents cell damage by providing sufficient energy [25, 26].

### 2.2 Mathematical model

The EBMM is a three‐state basic mathematical model proposed by R. N. Bergman for type 1 diabetes mellitus incorporating the effect of the meal disturbance during medication that is constant [27]. BGC is disturbed due to such meal disturbance, the state of which should be considered dynamical rather than static for accurate model behaviour [28]. The EBMM presented in [29] is an extension of Bergman's minimal model that incorporates meal disturbances as a state variable and is obtained by the following set of equations:

(1) $${\overset{\cdot }{x}}_{1}=-p_{1}\left(x_{1}-G_{b}\right)-x_{1}x_{2}+x_{4}$$

(2) $${\overset{\cdot }{x}}_{2}=-p_{2}x_{2}+p_{3}\left(x_{3}-I_{b}\right)$$

(3) $${\overset{\cdot }{x}}_{3}=-p_{4}\left(x_{3}-I_{b}\right)+u\left(t\right)$$

(4) $${\overset{\cdot }{x}}_{4}=-p_{5}x_{4}$$

where *x*_1_, *x*_2_, *x*_3_ and *x*_4_ are BGC, remote insulin concentration, plasma insulin concentration and meal disturbance, respectively, and *u*(*t*) is the control input law for external insulin infusion. The details of the other model parameters are given in Table 1 in Section 4.

### 2.3 Problem statement

**FIGURE 2:**
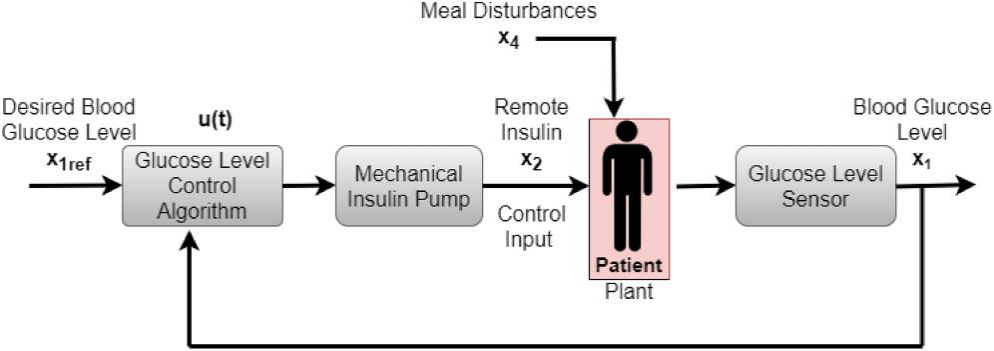
Closed‐loop control system for artificial pancreas

Keeping the BGC of type 1 diabetic patients in the normal range has always been a complex problem because it may be controlled by manual intake of insulin whenever the sugar level rises during daytime, but such control is not feasible during sleeping hours. During the night, there must be an automated system to control and regulate BGC in the patient body. AP helps to maintain and regulate BGC in diabetic patients by automatically injecting the required amount of insulin into the body. Precise information about BGC must be given to the AP, which uses control algorithms to infuse a controlled amount of insulin into the patient body. AP uses a sensor for this purpose, and the controller calculates the exact amount of insulin to inject and guides the insulin pump to inject that amount of insulin into the body of the patient. As the model given by Equations (1), (2), (3), (4) is non‐linear, designing a non‐linear controller can ensure global asymptotic stability. The proposed closed‐loop control scheme for an AP is shown in Figure 2.

## 3 ROBUST CONTROL ALGORITHMS DESIGN

### 3.1 Sliding mode‐controller design

**FIGURE 3:**
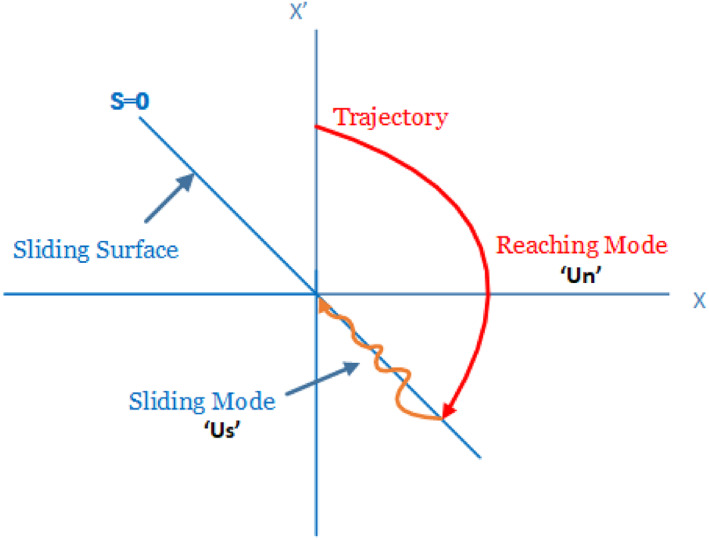
Phases of sliding mode controller

The SMC is robust against external disturbances for dynamical non‐linear systems. It should be designed with the aim that all dynamical states of the system should converge to the sliding surface *S* = 0 as shown by Figure 3.

In the SMC, the control law consists of two control parts; the nominal part *u*_*n*_ helps the trajectory of the system converge on the equilibrium point, while the switching control law *u*_*s*_ ensures that when the trajectory reaches the sliding surface, it is kept on the sliding surface until it falls at the origin. In the traditional SMC, the chattering phenomenon appears in the form of oscillations around the sliding surface due to the switching of *u*_*s*_ [30]. The overall control law can be defined as

$$u\left(t\right)=u_{n}+u_{s}$$

where

$$u_{n}=\left\{\begin{array}{cc} 1\to ON\; & \text{when}\;s>0\\ 0\to OFF\; & \text{when}\;s<0 \end{array}\right.$$

and

$$u_{s}=sign\left(s\right)$$

For the state variable *x*_1_ to track its desired blood glucose value, the error signal is defined as

(5) $$e_{1}=x_{1}-x_{1ref},$$

where *e*_1_ is the difference of BGC *x*_1_ to its reference value *x*_1*ref*_. To deal with the error signal given by Equation (5), the sliding surface for the SMC can be defined as

(6) $$\Gamma_{1}={\ddot{e}}_{1}+s_{1}{\overset{\cdot }{e}}_{1}+s_{0}e_{1}$$

where *s*_1_ and *s*_0_ are positive constants. By taking the time derivative of Γ_1_, we obtain

(7) $${\overset{\cdot }{\Gamma }}_{1}={\dddot{e}}_{1}+s_{1}{\ddot{e}}_{1}+s_{0}{\overset{\cdot }{e}}_{1}$$

Now computing the first, second and third derivatives, respectively, with regard to the time of Equation (5), we obtain

(8) $$\begin{array}{cc} {\overset{\cdot }{e}}_{1} & ={\overset{\cdot }{x}}_{1}-{\overset{\cdot }{x}}_{1ref}={\overset{\cdot }{x}}_{1}\\ {\ddot{e}}_{1} & ={\ddot{x}}_{1}-{\ddot{x}}_{1ref}={\ddot{x}}_{1}\\ {\dddot{e}}_{1} & ={\dddot{x}}_{1}-{\dddot{x}}_{1ref}={\dddot{x}}_{1} \end{array}$$

where *x*_1*ref*_ is constant, so ${\dddot{x}}_{1ref}=0$. By using Equation (8), ${\overset{\cdot }{\Gamma }}_{1}$ can be written as

(9) $${\overset{\cdot }{\Gamma }}_{1}={\dddot{x}}_{1}+s_{1}{\ddot{x}}_{1}+s_{0}{\overset{\cdot }{x}}_{1}$$

Now differentiating Equation (1) to the third derivative, we obtain

(10) $$\begin{array}{cc} {\dddot{x}}_{1} & =p_{1}^{2}{\overset{\cdot }{x}}_{1}+2p_{1}\left({\overset{\cdot }{x}}_{1}x_{2}+x_{1}{\overset{\cdot }{x}}_{2}\right)-p_{1}{\overset{\cdot }{x}}_{4}+p_{1}G_{b}{\overset{\cdot }{x}}_{2}+{\overset{\cdot }{x}}_{1}x_{2}^{2}\\ & +2x_{1}x_{2}{\overset{\cdot }{x}}_{2}-{\overset{\cdot }{x}}_{2}x_{4}-x_{2}{\overset{\cdot }{x}}_{4}+p_{2}\left({\overset{\cdot }{x}}_{1}x_{2}+x_{1}{\overset{\cdot }{x}}_{2}\right)-p_{3}\left({\overset{\cdot }{x}}_{1}x_{3}\right)\\ & -p_{3}x_{1}\left(-p_{4}\left(x_{3}-I_{b}\right)\right)-p_{3}x_{1}u\left(t\right)+p_{3}I_{b}{\overset{\cdot }{x}}_{1}-p_{5}{\overset{\cdot }{x}}_{4} \end{array}$$

If we denote

(11) $$\begin{array}{cc} ϒ\left(t\right) & =p_{1}^{2}{\overset{\cdot }{x}}_{1}+2p_{1}\left({\overset{\cdot }{x}}_{1}x_{2}+x_{1}{\overset{\cdot }{x}}_{2}\right)-p_{1}{\overset{\cdot }{x}}_{4}+p_{1}G_{b}{\overset{\cdot }{x}}_{2}+{\overset{\cdot }{x}}_{1}x_{2}^{2}\\ & +2x_{1}x_{2}{\overset{\cdot }{x}}_{2}-{\overset{\cdot }{x}}_{2}x_{4}-x_{2}{\overset{\cdot }{x}}_{4}+p_{2}\left({\overset{\cdot }{x}}_{1}x_{2}+x_{1}{\overset{\cdot }{x}}_{2}\right)\\ & -p_{3}\left({\overset{\cdot }{x}}_{1}x_{3}\right)-p_{3}x_{1}\left(-p_{4}\left(x_{3}-I_{b}\right)\right)+p_{3}I_{b}{\overset{\cdot }{x}}_{1}-p_{5}{\overset{\cdot }{x}}_{4} \end{array}$$

then Equation (10) becomes

(12) $${\dddot{x}}_{1}=ϒ\left(t\right)-p_{3}x_{1}u\left(t\right)$$

Using Equation (12) in Equation (33), we have

(13) $${\overset{\cdot }{\Gamma }}_{1}=ϒ\left(t\right)-p_{3}x_{1}u\left(t\right)+s_{1}{\ddot{x}}_{1}+s_{0}{\overset{\cdot }{x}}_{1}$$

To make ${\overset{\cdot }{\Gamma }}_{1}$ negative definite, we use

(14) $${\overset{\cdot }{\Gamma }}_{1}=-K|\Gamma_{1}{|}^{\alpha }sign\left(\frac{\Gamma_{1}}{\phi }\right)$$

where *K* is the positive design coefficient, and *ϕ* is the small number used to remove chattering and *α* is between 0 and 1, while |Γ_1_|^*α*^ ensures the convergence of the system trajectories to siding surface *Γ*_1_ = 0.

Comparing Equations (13) and (14) gives

(15) $$-K|\Gamma_{1}{|}^{\alpha }sign\left(\frac{\Gamma_{1}}{\phi }\right)=ϒ\left(t\right)-p_{3}x_{1}u\left(t\right)+s_{1}{\ddot{x}}_{1}+s_{0}{\overset{\cdot }{x}}_{1}$$

Consider a positive definite Lyapunov candidate function as

(16) $$V_{1}=\frac{1}{2}\Gamma_{1}^{2}$$

Differentiating Equation (16) with respect to time yields

(17) $${\overset{\cdot }{V}}_{1}=\Gamma_{1}{\overset{\cdot }{\Gamma }}_{1}$$

Using the value of ${\overset{\cdot }{\Gamma }}_{1}$ from Equation (14) obtains

(18) $${\overset{\cdot }{V}}_{1}=\Gamma_{1}\left(-K|\Gamma_{1}{|}^{\alpha }sign\left(\frac{\Gamma_{1}}{\phi }\right)\right)$$

as

(19) $$\frac{\Gamma_{1}}{\phi }sign\left(\frac{\Gamma_{1}}{\phi }\right)=\left|\frac{\Gamma_{1}}{\phi }\right|$$

so Equation (18) becomes

(20) $${\overset{\cdot }{V}}_{1}=-K|\Gamma_{1}{|}^{\alpha }\phi \left|\frac{\Gamma_{1}}{\phi }\right|=-K|\Gamma_{1}{|}^{\alpha }\phi \frac{\left|\Gamma_{1}\right|}{\phi }$$

and because |*ϕ*| = *ϕ* and *ϕ* > 0, we have

(21) $${\overset{\cdot }{V}}_{1}=-K|\Gamma_{1}{|}^{\alpha +1}$$

So, the time derivative of the Lyapunov candidate function ${\overset{\cdot }{V}}_{1}$ given by Equation (21) is proved to be negative definite. Hence, the system is globally asymptotically stable.

Rewriting Equation (15) and solving it, we obtain the control input *u*(*t*) as

(22) $$u\left(t\right)=\frac{1}{p_{3}x_{1}}\left[ϒ\left(t\right)+s_{1}{\ddot{x}}_{1}+s_{0}{\overset{\cdot }{x}}_{1}+K|\Gamma_{1}{|}^{\alpha }sign\left(\frac{\Gamma_{1}}{\phi }\right)\right]$$

where nominal control *u*_*n*_ is

$$u_{n}=\frac{1}{p_{3}x_{1}}\left[ϒ\left(t\right)+s_{1}{\ddot{x}}_{1}+s_{0}{\overset{\cdot }{x}}_{1}\right]$$

and switching control *u*_*s*_ is

$$u_{s}=\frac{1}{p_{3}x_{1}}\left[K|\Gamma_{1}{|}^{\alpha }sign\left(\frac{\Gamma_{1}}{\phi }\right)\right]$$

The controller *u*(*t*) given by Equation (22) is the required control input to make the system track BGC to its reference value using the first‐order SMC. Since the state variable *x*_1_ represents BGC, it is supposed to be a higher value, and the proposed controller brings it down to the safe range of 70–120 mg/dl. It always remains a positive value and never reaches zero because BGC at zero means the death of a patient, which restricts the control input from becoming infinite.

Now the SMC in case of disturbance/noise can be analysed by adding Gaussian noise *d*(*t*) in Equation (1) of the system as follows:

(23) $${\overset{\cdot }{x}}_{1n}=\left[-p_{1}\left(x_{1}-G_{b}\right)-x_{1}x_{2}+x_{4}\right]+d\left(t\right)$$

where *d*(*t*) satisfies the following inequality:

(24) d(t)≤K

where *K* is the value of design coefficient used in the control input. The sliding surface for the SMC in case of noise is same as defined by Equation (6) while the error signal can be written as

(25) $$e_{1n}=x_{1n}-x_{1refn}$$

where *x*_1*n*_ is the BGC in the presence of noise and *x*_1*refn*_ is the reference value with noise. Now by repeating the same process as done above for designing the SMC, the value of control input *u*(*t*) in the presence of external disturbance can be defined as

(26) $$u\left(t\right)=\frac{1}{p_{3}x_{1}}\left[ϒ\left(t\right)+s_{1}{\ddot{x}}_{1n}+s_{0}{\overset{\cdot }{x}}_{1n}+K|\Gamma_{1}{|}^{\alpha }sign\left(\frac{\Gamma_{1}}{\phi }\right)\right]$$

### 3.2 Supertwisting controller design

The conventional SMC results in inherent chattering phenomenon which is countered by the supertwisting SMC algorithm. The supertwisting SMC is capable of twisting all the system trajectories around the origin in finite time which gives chattering free convergence more rapidly. The BGC of type 1 diabetic patient needs to be kept in the safe range by controlling high BGC so, for such control problem the error signal for BGC can be written as

(27) $$e_{2}=x_{1}-x_{1ref}$$

The systems with relative degree of one can be continuously controlled by the supertwisting SOSMC such that the error signal must approaches to zero. To get relative degree equals to one, the sliding surface for supertwisting SOSMC can be defined as

(28) $$\Gamma_{2}={\ddot{e}}_{2}+s_{3}{\overset{\cdot }{e}}_{2}+s_{2}e_{2}$$

where *s*_2_ and *s*_3_ are real constants. The Lyapunov candidate function for the sliding surface Γ_2_ given by Equation (28) can be written as

(29) $$V_{2}=\frac{1}{2}\Gamma_{2}^{2}$$

Differentiating Equation (29) with respect to time yields

(30) $${\overset{\cdot }{V}}_{2}=\Gamma_{2}{\overset{\cdot }{\Gamma }}_{2}$$

By taking time derivative of the sliding surface given by Equation (28), we have

(31) $${\overset{\cdot }{\Gamma }}_{2}={\dddot{e}}_{2}+s_{3}{\ddot{e}}_{2}+s_{2}{\overset{\cdot }{e}}_{2}$$

Now computing the first, second and third derivative, respectively, with regard to the time of Equation (27), we obtain

(32) $$\begin{array}{cc} {\overset{\cdot }{e}}_{2} & ={\overset{\cdot }{x}}_{1}-{\overset{\cdot }{x}}_{1ref}={\overset{\cdot }{x}}_{1}\\ {\ddot{e}}_{2} & ={\ddot{x}}_{1}-{\ddot{x}}_{1ref}={\ddot{x}}_{1}\\ {\dddot{e}}_{2} & ={\dddot{x}}_{1}-{\dddot{x}}_{1ref}={\dddot{x}}_{1} \end{array}$$

where *x*_1*ref*_ is constant, so ${\dddot{x}}_{1ref}=0$. By using Equation (32), ${\overset{\cdot }{\Gamma }}_{2}$ can be written as

(33) $${\overset{\cdot }{\Gamma }}_{2}={\dddot{x}}_{1}+s_{3}{\ddot{x}}_{1}+s_{2}{\overset{\cdot }{x}}_{1}$$

Inserting the value of ${\dddot{x}}_{1}$ from Equation (12) into Equation (33), we obtain

(34) $${\overset{\cdot }{\Gamma }}_{2}=ϒ\left(t\right)-p_{3}x_{1}u\left(t\right)+s_{3}{\ddot{x}}_{1}+s_{2}{\overset{\cdot }{x}}_{1}$$

The supertwisting SOSMC comprises the two control laws *u*_*n*_ and *u*_*s*_ as elaborated by Figure 3. The nominal control law *u*_*n*_ can be obtained from Equation (34) as

(35) $$u_{n}=\frac{1}{p_{3}x_{1}}\left(ϒ\left(t\right)+s_{3}{\ddot{x}}_{1}+s_{2}{\overset{\cdot }{x}}_{1}\right)$$

The phenomenon of chattering is observed in the traditional SMC in the form of oscillations around the sliding surface. The supertwisting SOSMC can deal with chattering by filtering out most of this phenomenon by having the second‐order SMC control law. The non‐linear first‐order differential equation can be defined as [31]

(36) $$g\left(t\right)={\overset{\cdot }{\Gamma }}_{2}+\beta_{1}|\Gamma_{2}{|}^{0.5}+\beta_{2}\int sign\left(\Gamma_{2}\right)dt$$

where *β*_1_, *β*_2_ > 0, and

$$sign\left(\Gamma_{2}\right)=\left\{\begin{array}{cc} -1\; & \text{when}\;\Gamma_{2}<0\\ 1\; & \text{when}\;\Gamma_{2}>0 \end{array}\right.$$

The first derivative and solution of Equation (36) will converges to zero in finite time if the design parameters β_1_ ≥ 0.5(*T*)_0.5_, β_2_ ≥ 4*T* and |*g*(*t*)| ≤ *T*, where *T* is the real positive number [32]. The switching control law for supertwisting SOSMC can be defined as [33]

(37) $$u_{s}=\frac{1}{p_{3}x_{1}}\left(\beta_{1}|\Gamma_{2}{|}^{0.5}sign\left(\Gamma_{2}\right)+\beta_{2}\int sign\left(\Gamma_{2}\right)dt\right)$$

By combining the nominal and switching control laws from Equations (35) and (37), respectively, we have the final control law *u*(*t*) as

(38) $$u\left(t\right)=\frac{1}{p_{3}x_{1}}[ϒ\left(t\right)+s_{3}{\ddot{x}}_{1}+s_{2}{\overset{\cdot }{x}}_{1}+\beta_{1}|\Gamma_{2}{|}^{0.5}sign\left(\Gamma_{2}\right)+\beta_{2}\int sign\left(\Gamma_{2}\right)dt]$$

Now the time derivative of the Lyapunov candidate function, which is ${\overset{\cdot }{V}}_{2}$ from Equation (30), can be updated using Equation (34) in it, and we obtain

(39) $${\overset{\cdot }{V}}_{2}=\Gamma_{2}\left(ϒ\left(t\right)-p_{3}x_{1}u\left(t\right)+s_{3}{\ddot{x}}_{1}+s_{2}{\overset{\cdot }{x}}_{1}\right)$$

By using *u*(*t*) from Equation (38) in Equation (39), the expression of ${\overset{\cdot }{V}}_{2}$ can be written as

(40) $${\overset{\cdot }{V}}_{2}=-\beta_{1}|\Gamma_{2}{|}^{0.5}|\Gamma_{2}|-\beta_{2}\int sign\left.\left(\Gamma_{2}\right)dt\right)$$

Hence, the time derivative of the Lyapunov candidate function ${\overset{\cdot }{V}}_{2}$ is proved to be negative definite, which means that the supertwisting SOSMC will ensure convergence of BGC to the reference level *x*_1ref_ in finite time. Consequently, the error signal *e*_2_ for BGC approaches zero even in the presence of all external disturbances. As ${\overset{\cdot }{V}}_{2}$ is negative definite, so global asymptotic stability of the system is ensured.

Now the supertwisting SOSMC in the case of disturbance/noise is analysed in the presence of Gaussian noise *d*(*t*) in the state variable *x*_1_. Considering Equations (23), Equation (24) and the sliding surface the same as Γ_2_, the error signal can be written as

(41) $$e_{2n}=x_{1n}-x_{1refn}$$

where *x*_1*n*_ is the BGC in the presence of noise, and *x*_1*refn*_ is the reference value with noise. Now by repeating the same process as above for designing the supertwisting SOMC, the value of control input *u*(*t*) in the presence of external disturbance can be defined as

(42) $$\begin{array}{cc} u\left(t\right) & =\frac{1}{p_{3}x_{1}}\left[ϒ\left(t\right)+s_{3}{\ddot{x}}_{1n}+s_{2}{\overset{\cdot }{x}}_{1n}+\beta_{1}|\Gamma_{2}{|}^{0.5}sign\left(\Gamma_{2}\right)\right.\\ & +\left.\beta_{2}\int sign\left(\Gamma_{2}\right)dt\right] \end{array}$$

### 3.3 Backstepping sliding mode controller design

Strict feedback from the system is required to derive the controller expression by defining error functions for all the state variables and proving them negative definite using Lyapunov stability theory. BS is a recursive technique that gives the asymptotic stability of the system. To enhance the performance of BS and add robustness, the SMC is merged with the BS algorithm. The error *z*_1_ for tracking of BGC in the presence of Gaussian noise is defined as

(43) $$z_{1}=x_{1n}-x_{1refn}$$

When the BGC *x*_1*n*_ tracks the reference value *x*_1*refn*_, the error *z*_1_ converges to zero. By taking the time derivative of *z*_1_ and using Equation (23), we have

(44) $$\begin{array}{cc} {\overset{\cdot }{z}}_{1} & ={\overset{\cdot }{x}}_{1n}-{\overset{\cdot }{x}}_{1refn}\\ {\overset{\cdot }{z}}_{1} & =\left[-p_{1}\left(x_{1}-G_{b}\right)-x_{1}x_{2}+x_{4}\right]+d\left(t\right)-{\overset{\cdot }{x}}_{1refn} \end{array}$$

The Lyapunov function candidate to analyse the stability of *z*_1_ is defined as

(45) $$V_{1}=\frac{1}{2}z_{1}^{2}$$

To get asymptotic stability, the time derivative of *V*_1_ must be proved to be negative definite. Now by computing the time derivative of *V*_1_, we have

(46) $${\overset{\cdot }{V}}_{1}=z_{1}{\overset{\cdot }{z}}_{1}$$

The error for the state variable *x*_2_ can be defined as

(47) $$z_{2}=x_{2}-\sigma_{2}$$

where σ_2_ is the first virtual control law. We can also write Equation (47) as

(48) $$x_{2}=z_{2}+\sigma_{2}$$

When the state variable *x*_2_ tracks *σ*_2_, the error *z*_2_ converges to zero. By substituting *x*_2_ from Equation (48) in Equation (44), we obtain

(49) $${\overset{\cdot }{z}}_{1}=\left[-p_{1}\left(x_{1}-G_{b}\right)-x_{1}\left(z_{2}+\sigma_{2}\right)+x_{4}\right]+d\left(t\right)-{\overset{\cdot }{x}}_{1refn}$$

Now by using ${\overset{\cdot }{z}}_{1}$ from Equation (49) in Equation (46), ${\overset{\cdot }{V}}_{1}$ can be written as

(50) $${\overset{\cdot }{V}}_{1}=\left[z_{1}\left(-p_{1}\left(x_{1}-G_{b}\right)-x_{1}\left(z_{2}+\sigma_{2}\right)+x_{4}\right]+d\left(t\right)-{\overset{\cdot }{x}}_{1refn}\right)$$

The prove that the virtual control law σ_2_ ensures that ${\overset{\cdot }{V}}_{1}$ is negative definite, we put

(51) $$\left[-p_{1}\left(x_{1}-G_{b}\right)-x_{1}\left(z_{2}+\sigma_{2}\right)+x_{4}\right]+dt-{\overset{\cdot }{x}}_{1refn}=-k_{1}z_{1}$$

where *k*_1_ is a positive constant. The virtual control σ_2_ from Equation (51) can be defined as

(52) $$\sigma_{2}=\frac{1}{\left(x_{1}\right)}\left(\left[-p_{1}\left(x_{1}-G_{b}\right)-x_{1}z_{2}+x_{4}\right]+d\left(t\right)-{\overset{\cdot }{x}}_{1refn}+k_{1}z_{1}\right)$$

By using the value of σ_2_ in Equation (50), we have

(53) $${\overset{\cdot }{V}}_{1}=-k_{1}z_{1}^{2}-z_{1}z_{2}x_{1}$$

To check convergence of both the errors *z*_1_ and *z*_2_ to zero, we take the second Lyapunov candidate function as

(54) $$V_{2}=V_{1}+\frac{1}{2}z_{2}^{2}$$

By computing time derivative of *V*_2_, we have

(55) $${\overset{\cdot }{V}}_{2}={\overset{\cdot }{V}}_{1}+z_{2}{\overset{\cdot }{z}}_{2}$$

By taking time derivative of Equation (47) and using Equation (2), we have

(56) $$\begin{array}{cc} {\overset{\cdot }{z}}_{2} & ={\overset{\cdot }{x}}_{2}-{\overset{\cdot }{\sigma }}_{2}\\ {\overset{\cdot }{z}}_{2} & =-p_{2}x_{2}+p_{3}\left(x_{3}-I_{b}\right)-{\overset{\cdot }{\sigma }}_{2} \end{array}$$

To introduce the SMC in the BS algorithm, the sliding surface variable Γ_3_ can be defined as

(57) $$\Gamma_{3}=x_{3}-\sigma_{3}$$

where σ_3_ is the second virtual control law. We can also write Equation (57) as

(58) $$x_{3}=\Gamma_{3}+\sigma_{3}$$

When the state variable *x*_3_ tracks σ_3_, the error Γ_3_ converges to zero. By substituting *x*_3_ from Equation (58) in Equation (56), we obtain

(59) $${\overset{\cdot }{z}}_{2}=-p_{2}x_{2}+p_{3}\left(\Gamma_{3}+\sigma_{3}\right)-p_{3}I_{b}-{\overset{\cdot }{\sigma }}_{2}$$

Now by using ${\overset{\cdot }{V}}_{1}$ from Equation (53) and ${\overset{\cdot }{z}}_{2}$ from Equation (59) in Equation (55), respectively, ${\overset{\cdot }{V}}_{2}$ can be written as

(60) $$\begin{array}{cc} {\overset{\cdot }{V}}_{2} & =-k_{1}z_{1}^{2}-z_{1}z_{2}x_{1}+z_{2}\left(-p_{2}x_{2}+p_{3}\left(\Gamma_{3}+\sigma_{3}-I_{b}\right)-{\overset{\cdot }{\sigma }}_{2}\right)\\ {\overset{\cdot }{V}}_{2} & =-k_{1}z_{1}^{2}+z_{2}\left(-p_{2}x_{2}+p_{3}\left(\Gamma_{3}+\sigma_{3}-I_{b}\right)-{\overset{\cdot }{\sigma }}_{2}-z_{1}x_{1}\right) \end{array}$$

To prove that the virtual control law *σ*_3_ ensures that $\overset{\cdot }{V_{2}}$ is negative definite, we put

(61) $$-p_{2}x_{2}+p_{3}\left(\Gamma_{3}+\sigma_{3}-I_{b}\right)-{\overset{\cdot }{\sigma }}_{2}-z_{1}x_{1}=-k_{2}z_{2}$$

where *k*_2_ is a positive constant. The virtual control σ_3_ from Equation (61) can be defined as

(62) $$\sigma_{3}=\frac{1}{p_{3}}\left(-k_{2}z_{2}+p_{2}x_{2}-p_{3}\left(\Gamma_{3}-I_{b}\right)+{\overset{\cdot }{\sigma }}_{2}+z_{1}x_{1}\right)$$

By using the value of σ_3_ in Equation (60), we have

(63) $${\overset{\cdot }{V}}_{2}=-k_{1}z_{1}^{2}-k_{2}z_{2}^{2}+z_{2}\Gamma_{3}p_{3}$$

By taking time derivative of the sliding surface *S* from Equation (57) and using Equation (3), we have

(64) $$\begin{array}{cc} {\overset{\cdot }{\Gamma }}_{3} & ={\overset{\cdot }{x}}_{3}-{\overset{\cdot }{\sigma }}_{3}\\ {\overset{\cdot }{\Gamma }}_{3} & =-p_{4}\left(x_{3}-I_{b}\right)+u-{\overset{\cdot }{\sigma }}_{3} \end{array}$$

To check the convergence of the errors *z*_1_, *z*_2_ and the sliding surface Γ_3_ to zero, we take the composite Lyapunov candidate function as

(65) $$V_{3}=V_{2}+\frac{1}{2}\Gamma_{3}^{2}$$

By computing time derivative of *V*_3_, we have

(66) $${\overset{\cdot }{V}}_{3}={\overset{\cdot }{V}}_{2}+\Gamma_{3}{\overset{\cdot }{\Gamma }}_{3}$$

Now by using ${\overset{\cdot }{V}}_{2}$ from Equation (63) and ${\overset{\cdot }{\Gamma }}_{3}$ from Equation (64) respectively, $\overset{\cdot }{V_{3}}$ can be written as

(67) $$\begin{array}{cc} {\overset{\cdot }{V}}_{3} & =-k_{1}z_{1}^{2}-k_{2}z_{2}^{2}+z_{2}\Gamma_{3}p_{3}+\Gamma_{3}\left(-p_{4}\left(x_{3}-I_{b}\right)+u-{\overset{\cdot }{\sigma }}_{3}\right)\\ {\overset{\cdot }{V}}_{3} & =-k_{1}z_{1}^{2}-k_{2}z_{2}^{2}+\Gamma_{3}\left(z_{2}p_{3}-p_{4}\left(x_{3}-I_{b}\right)+u-{\overset{\cdot }{\sigma }}_{3}\right) \end{array}$$

The control law *u*(*t*) given by Equation (68) is the required BSMC controller that makes ${\overset{\cdot }{V}}_{3}$ negative definite and is given by

(68) $$u\left(t\right)=-k_{3}\Gamma_{3}-z_{2}p_{3}+p_{4}\left(x_{3}-I_{b}\right)+{\overset{\cdot }{\sigma }}_{3}-Ksign\left(\Gamma_{3}\right)$$

where *k*_3_ is a positive constant and the nominal control *u*_*n*_ is

$$u_{n}=k_{3}\Gamma_{3}-z_{2}p_{3}+p_{4}\left(x_{3}-I_{b}\right)+{\overset{\cdot }{\sigma }}_{3}$$

and switching control *u*_*s*_ is

$$u_{s}=-Ksign\left(\Gamma_{3}\right)$$

By using actual control law *u*(*t*) from Equation (68) in Equation (67), we have

(69) $${\overset{\cdot }{V}}_{3}=-k_{1}z_{1}^{2}-k_{2}z_{2}^{2}-k_{3}\Gamma_{3}^{2}$$

Hence, the time derivative of composite Lyapunov candidate function ${\overset{\cdot }{V}}_{3}$ for the errors *z*_1_, *z*_2_ and the sliding surface Γ_3_ is proved negative definite which ensures asymptotic stability of the system as all the errors converge to zero and BGC *x*_1_ tracks the reference value.

## 4 SIMULATION RESULTS

The proposed SMC, supertwisting SOSMC and SMC BS controllers given by Equations (26), (38) and (68), respectively, using EBMM have been simulated in the MATLAB/Simulink environment for observing their comparative performance for the regulation of the BGC in a type 1 diabetic patient. The horizontal *x*‐axis represents time (seconds), while the vertical *y*‐axis represents BGC (mg/dl). The safe range for BGC is 70–120 mg/dl, and for the tracking of BGC, the reference level *x*_1ref_ = 80 mg/d*l* is considered. The parametric values used for the simulation results are detailed in Table 1. The same parametric values are used in [34], and the same data set has been chosen for each simulation in this article because the results are comparable when the same data set is used for comparing them with each other.

**TABLE 1:** Non‐linear model parameters

Model parameters and descriptions		
Parameters	Parameter description	Parameter values
*p*_1_	Glucose effectiveness	0 min^−1^
*p*_2_	Insulin action delay	0.015 min^−1^
*p*_3_	Patient parameter	0.13 × 10^−4^ mUl^−1^ min^−2^
*p*_4_	Insulin decline rate	0.021 min^−1^
*p*_5_	Meal disturbance	0.05 min^−1^
*I*_*b*_	Plasma insulin basal	7 mUl^−1^
*G*_*b*_	Plasma glucose basal	80 mg dl^−1^

**TABLE 2:** Performance comparison of controllers

Comparison of proposed controllers			
Controller	Convergence time (min)	Steady‐state error	Chattering
Supertwisting SOSMC	6.66 or less	No	No
BSMC	6.67	No	No
SMC	15	No	Yes
PID	20.83 or greater	Yes	Yes

Abbreviations: BSMC, backstepping SMC; PID, proportional–integral–derivative; SMC, sliding mode controller; SOSMC, second‐order SMC.

**FIGURE 4:**
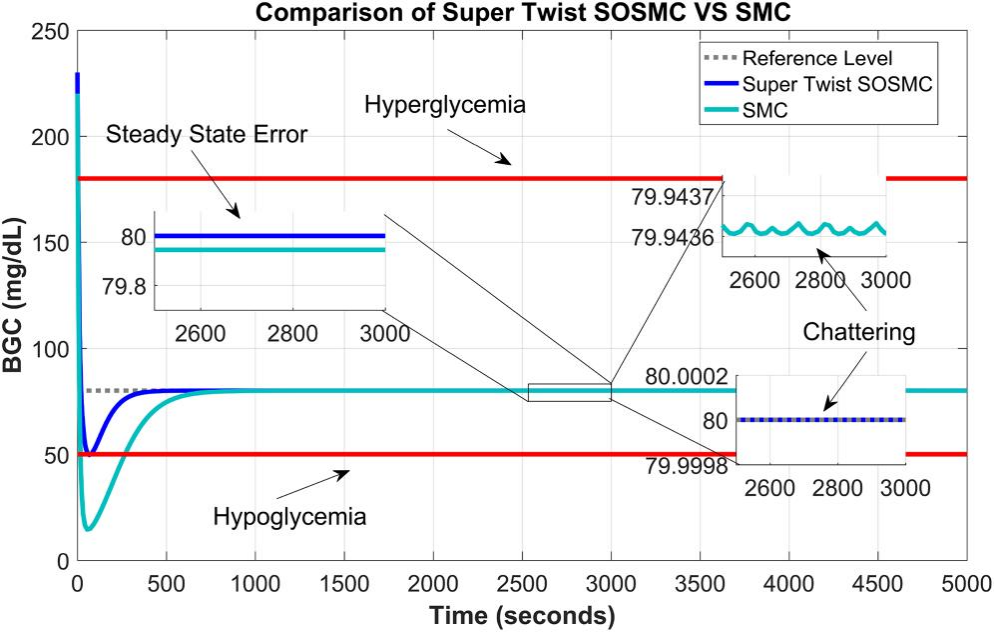
Comparison of supertwisting second‐order sliding mode controller and sliding mode controller

Figure 4 has been drawn for the comparative performance of the supertwisting SOSMC and SMC for tracking BGC. It can be observed from the graph that the SMC undergoes a larger undershoot and takes more time to settle down in steady state, whereas the supertwisting SOSMC gives better tracking response with a negligibly small undershoot and negligible chattering phenomenon.

**FIGURE 5:**
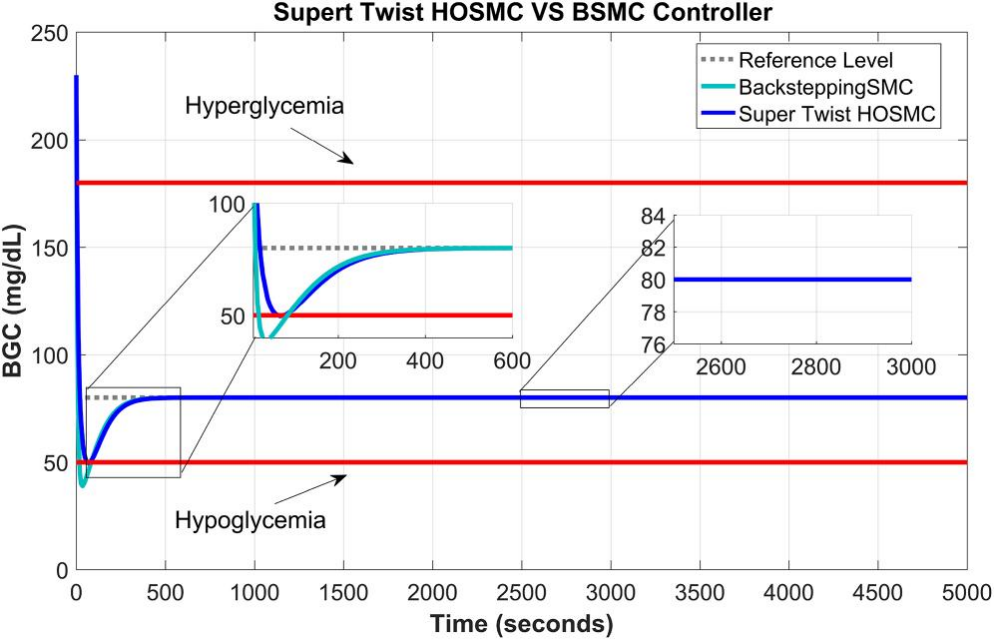
Comparison of supertwisting second‐order sliding mode controller and backstepping sliding mode controller

Figure 5 shows the comparison of the supertwisting SOSMC and BSMC. From the graph it can be observed that the supertwisting SOSMC and BSMC have nearly similar times of convergence, but the BSMC undergoes slightly larger chattering than that of the proposed supertwisting SOSMC. Both track the reference value quite nicely.

**FIGURE 6:**
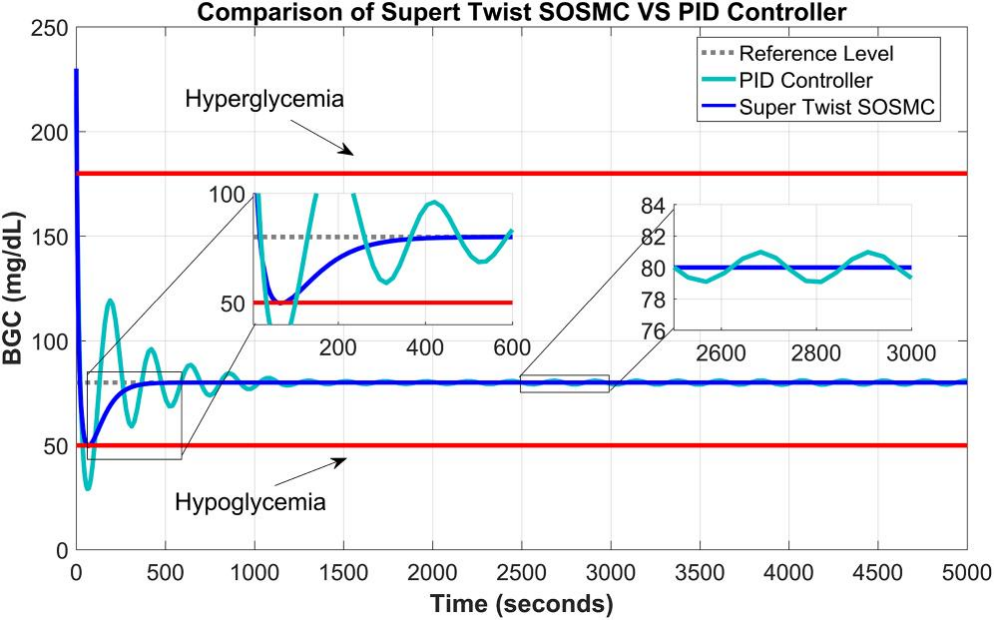
Comparison of supertwisting second‐order sliding mode controller with proportional–integral–derivative controller

A comparison of the supertwisting SOSMC and PID controller is made in Figure 6, which shows that the PID undergoes an oscillatory response with larger undershoots/overshoots, has very large settling time and has some steady‐state error in comparison with the supertwisting SOSMC. So it can be clearly observed that the performance of the PID controller is not satisfactory when compared with the supertwisting SOSMC in terms of oscillations, steady‐state error, undershoots/overshoots and convergence time.

**FIGURE 7:**
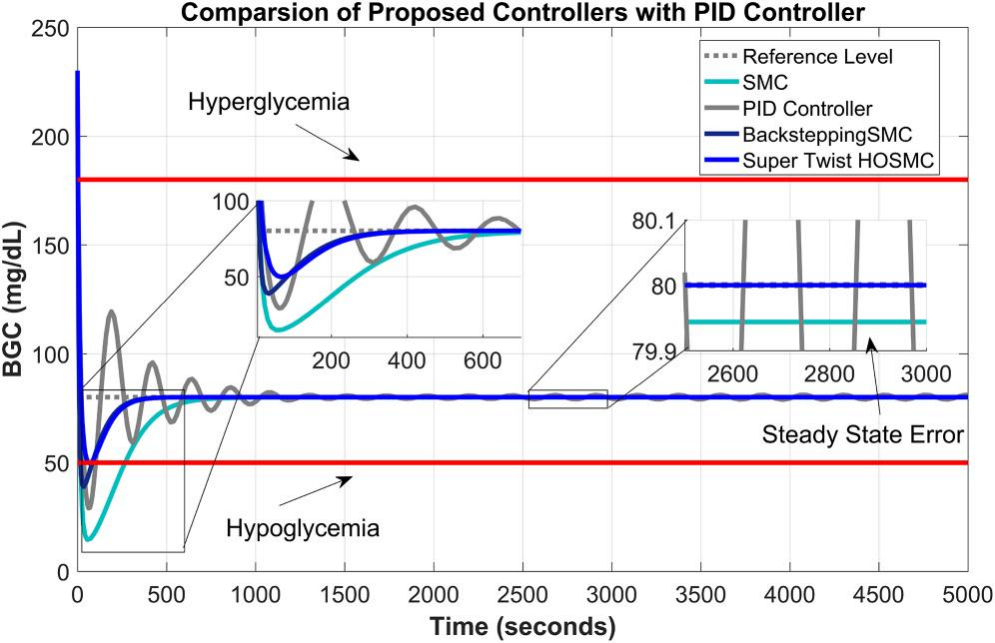
Comparison of proposed second‐order sliding mode, backstepping sliding mode, and supertwisting second‐order sliding mode controllers with proportional–integral–derivative controller

Comparisons of all the proposed controllers are given in Figure 7 for their comparative behaviour. It can be observed clearly from the graph that the supertwisting SOSMC performs better, with a convergence time of 6.66 min and no chattering phenomenon. The BSMC has a convergence time of 6.7 min at the expense of a slightly large undershoot. The SMC has convergence time of 15 min with larger undershoot and chattering phenomenon. The PID controller has oscillatory behaviour with larger undershoots/overshoots with convergence time of 20.83 min and also has some steady‐state error. The improvement made by the supertwisting SOSMC can be observed from its tracking response even in the presence of dynamical meal disturbances and Gaussian noise in view of all the comparison parameters. Hence, it can be deduced from the above performance comparison that the supertwisting SOSMC outperforms all the other proposed controllers in view of all performance evaluation parameters. A brief performance comparison of all the controllers under discussion is numerically detailed in Table 2.

**FIGURE 8:**
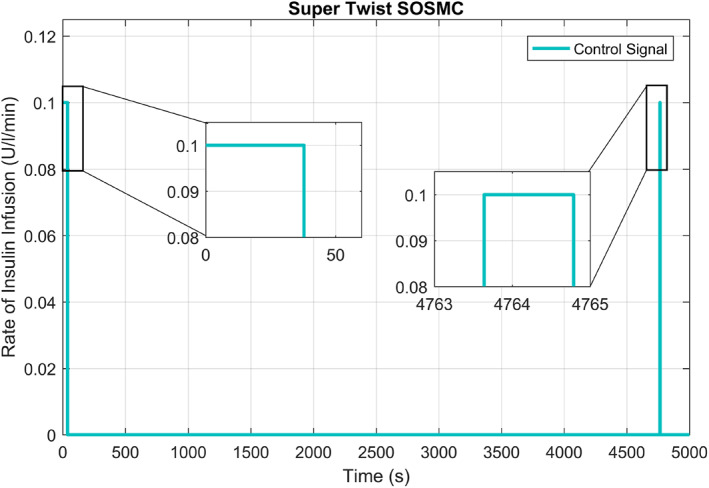
Control signal of supertwisting second‐order sliding mode controller

Figure 8 demonstrates the control input signal (which is the required amount of insulin to be injected into the patient body) by using the supertwisting SOSMC. To avoid over‐dosage of insulin infusion at different periods, the output of the controller is regulated by a saturation block in system response. The first pulse in the control signal causes the BGC to fall from higher to lower level, and then another pulse is injected by the controller to achieve the reference position. Then the output of the supertwisting SOSMC goes to zero when the BGC reaches the reference level of 80 mg/dl.

To observe the tracking response of the proposed supertwisting SOSMC under different parametric conditions, we have considered the data of six different type 1 diabetic patients available in the literature [35] mentioned in Tables 3 and 4.

**TABLE 3:** Patients’ parameter values (patients 1–3)

Patient data values			
System parameters	Patient 1	Patient 2	Patient 3
*p*_1_	0	0	0
*p*_2_	0.0107	0.0072	0.0142
*p*_3_	5.3* × *10^−6^	2.16* × *10^−6^	9.94* × *10^−6^
*p*_4_	0.264	0.2465	0.2814
*p*_5_	0.4	0.45	0.56
*G*_*b*_	80	80	80
*I*_*b*_	7	7	7
*G*_0_	220	200	180
*I*_*b*_	7	7	7
*I*_0_	50	55	60
*D*_0_	11.3	10	9.7

**TABLE 4:** Patients parameter values (patients 4–6)

System parameters	Patient 4	Patient 5	Patient 6
*p*_1_	0	0	0
*p*_2_	0.0083	0.0095	0.0230
*p*_3_	3.3* × *10^−6^	4.16* × *10^−6^	10.2* × *10^−6^
*p*_4_	0.273	0.310	0.3140
*p*_5_	0.49	0.50	0.59
*G*_*b*_	80	80	80
*I*_*b*_	7	7	7
*G*_0_	200	210	190
*I*_*b*_	7	7	7
*I*_0_	57	63	58
*D*_0_	9.9	9.2	10.8

**FIGURE 9:**
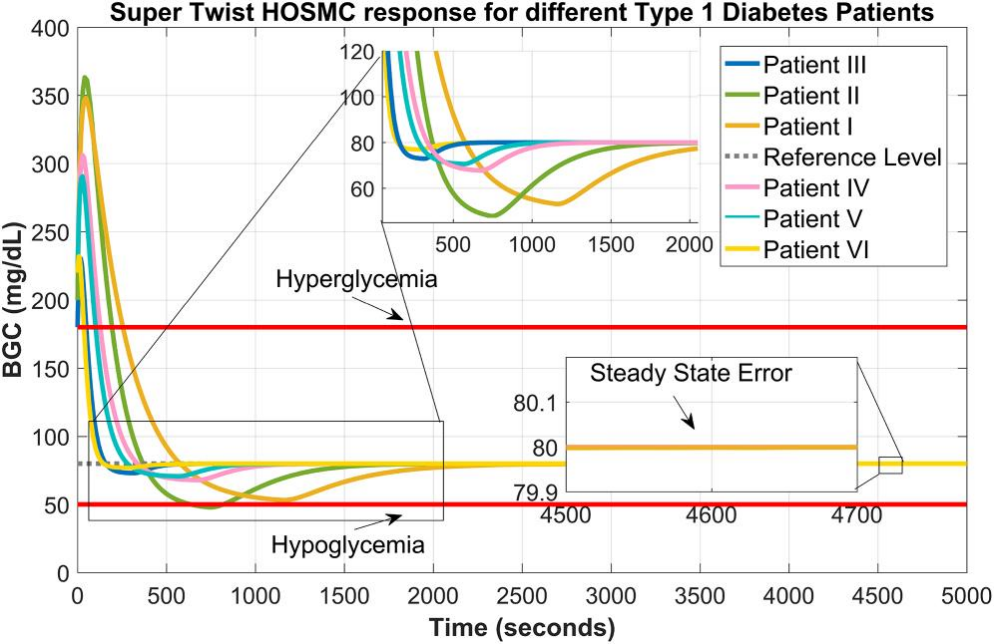
Tracking response of supertwisting second‐order sliding mode controller for various patients

Figure 9 shows that the supertwisting SOSMC effectively monitors and tracks the reference level of BGC for the data of three patients very nicely without undergoing chattering and steady‐state errors. This performance of the supertwisting SOSMC ensures that it can handle the parametric variations of data of different type 1 diabetic patients without exhibiting any effect in its response, which reflects its robustness for such conditions.

## 5 CONCLUSION

Herein, we have considered the EBMM for type 1 diabetic patients and proposed three non‐linear controllers—the SMC, supertwisting SOSMC and BSMC—for automatic stabilisation of BGC for AP. Global asymptotic stability of the proposed controllers has been proved using Lyapunov theory. The performance of each proposed controller has been analysed by the simulation results in MATLAB/Simulink in the presence of perturbation as Gaussian noise. It is observed from the results that the reference level is maintained perfectly by the proposed robust non‐linear controllers even in the presence of dynamical meal disturbances or burning sugar by physical exercise during medication. The proposed supertwisting SOSMC controller outperforms the SMC, BSMC and PID in terms of settling time, chattering, transients, under/overshoots and steady‐state error. In the future, the supertwisting SOSMC with parametric adaption can also be implemented to improve its response and get more robustness with the data of more type 1 diabetic patients.
